# Health in Adapted Youth Sports Study (HAYS): health effects of sports participation in children and adolescents with a chronic disease or physical disability

**DOI:** 10.1186/s40064-015-1589-z

**Published:** 2015-12-22

**Authors:** Kristel Lankhorst, Karin van der Ende-Kastelijn, Janke de Groot, Maremka Zwinkels, Olaf Verschuren, Frank Backx, Anne Visser-Meily, Tim Takken, On behalf of the HAYS study group

**Affiliations:** Research Group Lifestyle and Health, Institute of Human Movement Studies, University of Applied Sciences, Utrecht, The Netherlands; Partner of Shared Utrecht Pediatric Exercise Research (SUPER) Lab, Utrecht, The Netherlands; Child Development and Exercise Center, Wilhelmina Children’s Hospital, University Medical Center Utrecht, P.O. Box 85090, 3508AB Utrecht, The Netherlands; Brain Center Rudolf Magnus and Center of Excellence for Rehabilitation Medicine, University of Medical Center Utrecht and De Hoogstraat Rehabilitation, Utrecht, The Netherlands; Department of Rehabilitation, Nursing Science and Sports, University Medical Center Utrecht, Utrecht, The Netherlands

**Keywords:** Sports participation, Children, Physical fitness, Chronic disease, Physical disability, Health

## Abstract

**Background:**

In typically developing children, participation in sports has been proven to be positively correlated to both physical and psychosocial health outcomes. In children and adolescents with a physical disability or chronic disease participation in both recreational and competitive sports is often reduced, while for this population an active lifestyle may be even more important in reaching optimal levels of physical and psychosocial health. Therefore, the aim of the Health in Adapted Youth Sports (HAYS) Study is to determine both negative and positive effects of sports on children and adolescents with a chronic disease or physical disability.

**Methods:**

In this cross-sectional study differences will be compared in regards to physical and psychosocial health, cognitive functioning, school performance, daily physical activity and injuries between children and adolescents with a chronic disease or physical disability who participate in sports and those who do not. Children and adolescents, both ambulatory and wheelchair dependent, in the age of 10–19 years with a physical disability or chronic disease will be included. “Sports” is defined as participation in an organized sport at least two times a week for a duration of 3 months or more prior to the assessment. Parametric and non-parametric statistics will be used to determine the differences between the two groups.

**Discussion:**

This study provides insight in the effects of sports participation in relation to health, psychosocial functioning, physical activity and school performance in children and adolescents (10–19 years) with a chronic disease or physical disability. Results will guide healthcare professionals working with these children to better guide this population in reaching optimal levels of health and physical activity levels.

## Background

Children with disabilities often show reduced fitness levels and physical activity patterns and they participate less in competitive and recreational sports compared to their non-disabled peers (Murphy and Carbone 2008; van Brussel et al. 2011). The relationship between health and physical fitness has been studied by many authors.

In the general population, low level of physical activity is highly associated with low physical fitness, an increased cardiovascular and overall mortality (Arraiz et al. 1992; Blair et al. 1989; Colditz et al. 2002; Erikssen 2001). In typically developing children, participation in sports has been proven to be positively correlated to both physical and psychosocial health outcomes (Eime et al. 2013). Also for adults with various disabilities the physical, psychological, social, and economic benefits of participation in sports and recreational activities are described previously (Richter et al. 1996; Klapwijk 1987; Jackson and Davis 1983; Hutzter and Bar-Eli 1993). Because of the reduced fitness levels and physical activity pattern in children with a disability or chronic disease, support for an active lifestyle, including participation in sports, may be even more important in this population.

Next to the possible benefits of sports on health and fitness level, increasing evidence also shows benefits from physical activity on school performance and level of cognition could be influenced in a positive way. Children and adolescents without a medical condition are already known to perform better at school when being physically active (Basch 2011; Donnelly et al. 2009; Sallis et al. 1999; Singh et al. 2012). Childhood physical fitness is also associated with higher levels of cognition and appeared to be a good predictor of school performance and level of cognition 1 year later (Chaddock et al. 2011; Pontifex et al. 2012; Tomporowski et al. 2008; Chaddock et al. 2012; London and Castrechini 2011).

Participation in sports could also have an positive influence on health related quality of life (HRQoL) and self-worth (Eime et al. 2013). HRQoL refers to the impact of health and illness on an individual’s quality of life. In relation to sports, adult athletes with cerebral palsy for example reported a positive influence of sports participation on their HRQoL (Groff et al. 2009; Verschuren et al. 2007).

When looking at competence levels, Special Olympics participants with developmental disorders showed higher levels of self-worth and perceived physical competence in comparison to nonparticipants (Weiss et al. 2003). These findings might support the hypothesis that sports participation will enlarge the global self-worth of children and adolescents with a chronic disease and/or disability. But when motor competence is inadequate for the type of sport, a feeling of failure could predominate.

While there are many positive reasons for participating in sports or other physical activities for children with a chronic disease and/or disability, attention must be paid to the risk of acute and overuse injuries but also illness. Indeed parents and healthcare providers are wary of injuries due to participation in sports, which could further limit their child’s physical functioning. These worries are confirmed in healthy children. A recent study reported a higher absolute risk of getting injured and a high probability of sustaining the injury when adding physical education hours at school or increasing organized sports outside school (Adirim and Cheng 2003; Trifonov Rexen et al. 2014; World Health Organization 1991). At the same time though, the relative risk of injury seems higher for children with low levels of habitual physical activity (Bloemers et al. 2012). Studies also reported children with disabilities to have a higher risk of injury than children without disabilities, but these studies were limited to just one type of disability, sport, or injury (Sinclair and Xiang 2008). Identifying injury patterns and illness in children with disabilities is important to provide safety in sports activities and to prevent dropout in physical activities and return to an inactive lifestyle (Bloemers et al. 2012; Collard et al. 2009; Webborn et al. 2006).

**Fig. 1:**
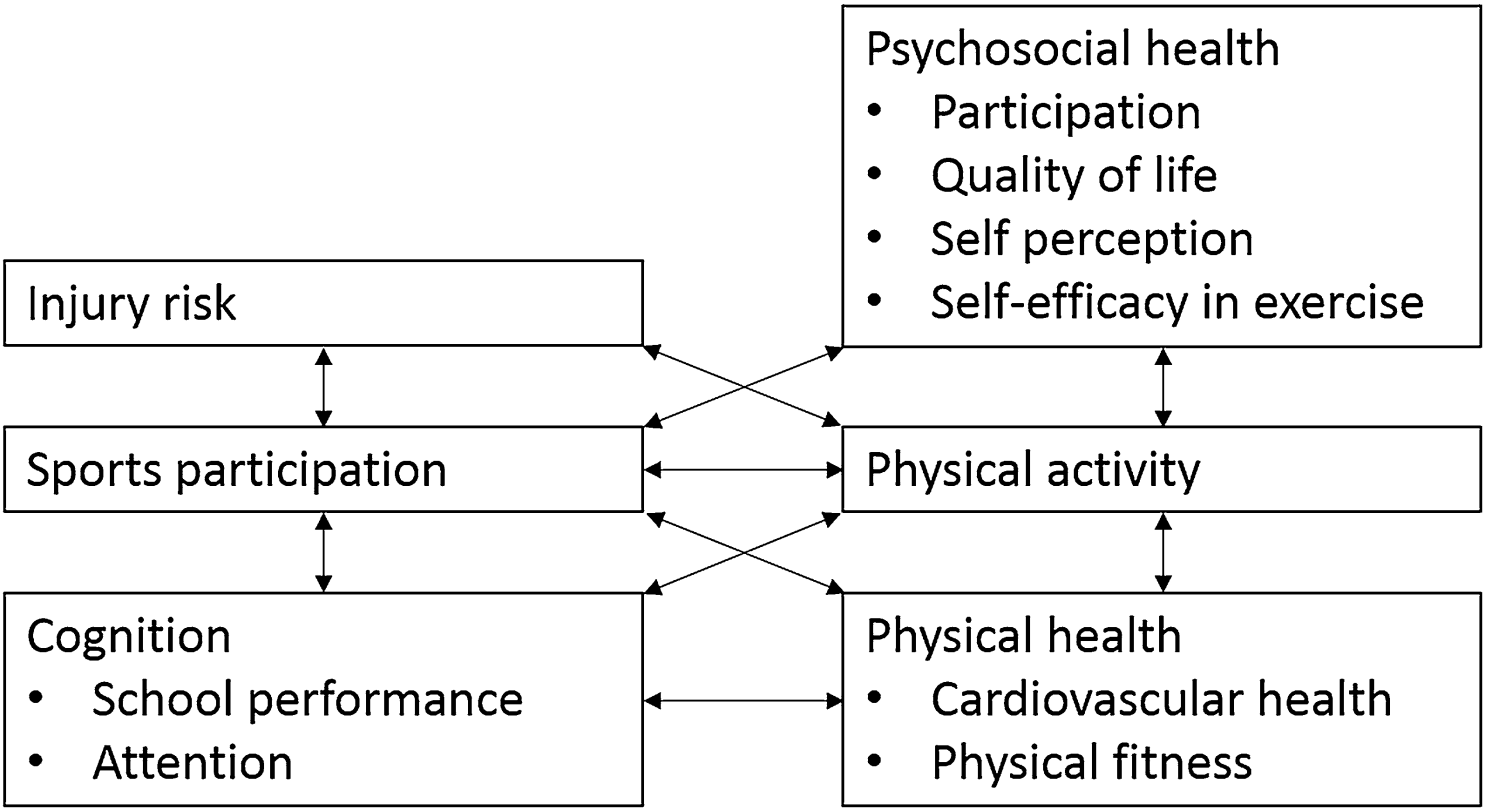
Overview of positive relations already established within a population of children and adolescents without a chronic disease and/or disability, and within a population of adults with and without a disability

In summary, children with chronic disease and/or disabilities often show reduced levels of physical activity and fitness and participate less in organized sports compared to their non-disabled peers. The positive effects of sports and daily activities on the psychosocial and physical health, cognition and injury risk as depicted in Fig. 1 already have been reported in a healthy population and disabled adults.

To date, however, limited evidence exists for these effects of participation in sports by children and adolescents with a physical disability or chronic disease. Therefore, the aim of the HAYS study is to determine both the positive and negative effects of sports related to health outcomes in children with disabilities and chronic childhood onset conditions. Therefore the Health in Adapted Youth Sports (HAYS) Study is designed to determine the positive and negative effects of participation in sports in this specific population.

## Design

**Fig. 2:**
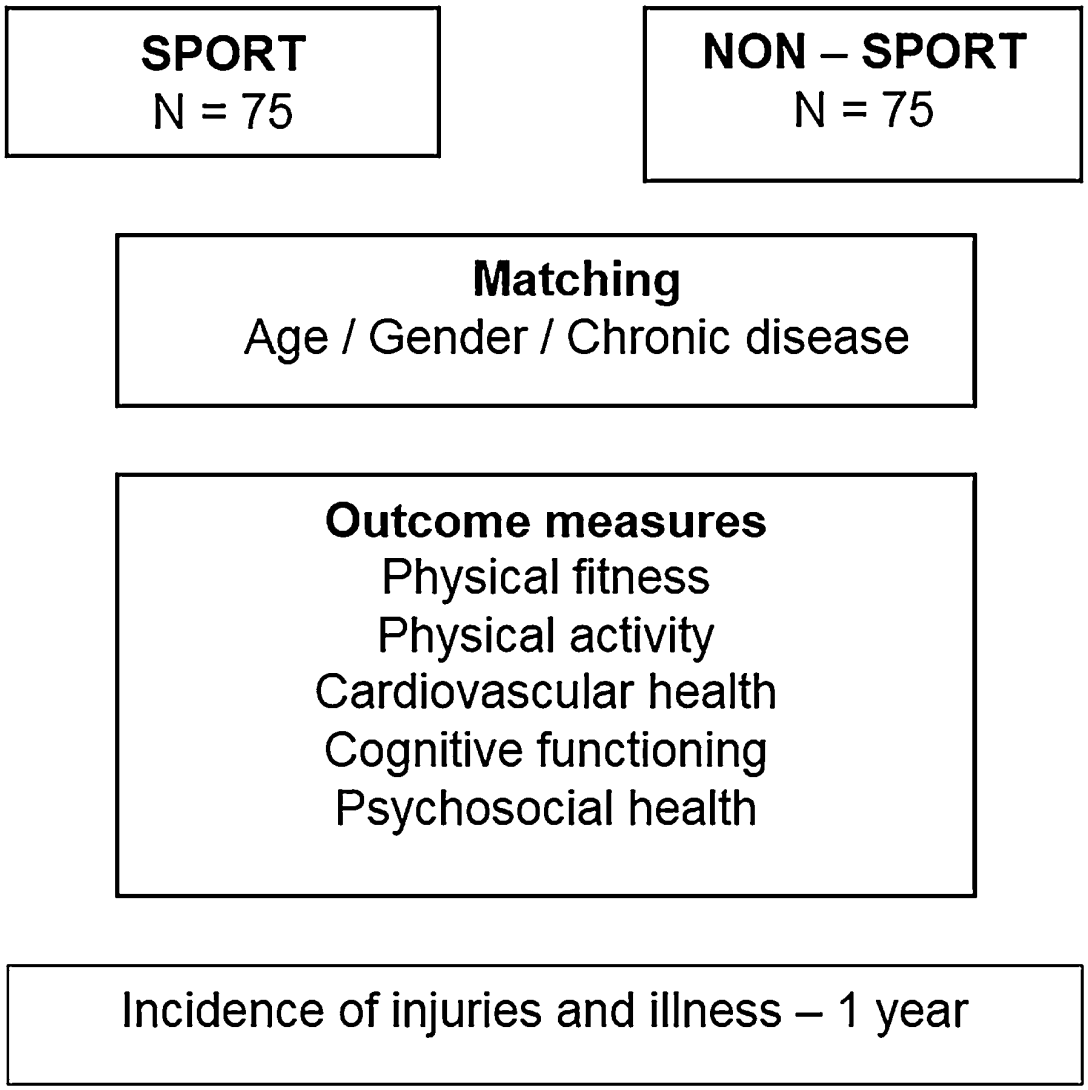
Overview of the design of the HAYS study

The HAYS study will be a cross-sectional study comparing children and adolescents with a physical disability or chronic disease, age 10–19 years, who are actively participating in organized sports to their non-sporting peers. The subjects who participate in sports will be matched on gender, age and diagnosis to their non-sporting peers, Fig. 2.

The current study is part of a larger project in which a controlled clinical trial will take place to evaluate the effectiveness of an after school sports program following a standardized interval training in children and adolescents with a chronic disease or physical disability. This study, titled The Sport-2-Stay-Fit study (S2SF; Trialregister.nl registration number: NTR4698), will use the same outcome instruments (Zwinkels et al. 2015).

### Participants

Eligible for this study are all children and adolescents aged from 10 up to 19 years with a physical chronic disease or condition, including cardiovascular, pulmonary, musculoskeletal, metabolic or neuromuscular disorders. Table 1 shows the in- and exclusion criteria. Both children who are ambulatory or those propelling a wheelchair are eligible for this study. Participants have to understand the Dutch language, understand simple instructions and be able to perform a physical fitness test. Children and adolescents in electric wheelchair, having a progressive disease or participate in other research projects which might influence the current study results will be excluded. Contra-indications for performing an exercise test, based on the exercise pre-participation screening questionnaire, may lead to exclusion on the cardiopulmonary exercise test (Balady et al. 1998).

**Table 1:** Eligibility and exclusion criteria

Eligibility	Exclusion
Children and adolescents with a physical disability or chronic disease: cardiovascular, pulmonary, musculoskeletal or neuromuscular disorder Children and adolescents between the age of 10 and 19 years Children and adolescents have to understand simple instructions Children and adolescents are able to perform physical fitness tests	Children and adolescents with progressive diseases Children and adolescents using an electric wheelchair During the length of the study, children are not allowed to participate in other research projects which might influence the current study results For the sporting group of the HAYS-study only: subjects who have not participated in any sports for the preceding 3 months No signed informed consent

Informed consent must be provided by all parents, as well as by subjects up from 12 years till 17 years. In line with Dutch law, no parental informed consent is required for subjects aged 18 years and over.

This study was approved by the Medical Ethics Committee of the University Medical Center Utrecht, the Netherlands.

### Recruitment

The children and adolescents will be recruited in the Netherlands among different patients associations, pediatric physical therapy practices, Wilhelmina Children’s Hospital in Utrecht, De Hoogstraat Rehabilitation Center in Utrecht, Fitkids, schools for children with a disability and sports clubs. Athletes will be recruited from a broad range of participation in sports: from recreational level to high level competitive sports.

### Setting

This study is a collaboration between the exercise lab at the University of Applied Sciences Utrecht, Wilhelmina Children’s Hospital and De Hoogstraat Rehabilitation Center in Utrecht, the Netherlands.

### Procedures

Criteria for eligibility and exclusion of subjects are depicted in Table 1. After permission of the subject and when the subject is eligible to participate in the study, the subject is scheduled for the assessment. Thereafter a secured link to four of the questionnaires will be sent to the participants or their parents by email in order to fill out the questionnaires online 1 week before the first testing moment. The researchers will assess the subjects subsequently, once on physical fitness, cognition, psychosocial- and cardiovascular health. Physical activity will be monitored during 1 week. The incidence of injuries and illness will be monitored for the duration of 1 year, by sending a secured link to a 5-item questionnaire every 2 weeks by email. If an injury or illness is reported, a telephone conversation will follow, to get insight and to classify the sort of injury or illness. Table 2 shows the procedure for the principle researchers and the subjects in the HAYS study, from recruitment to the end of participation.

**Table 2:**
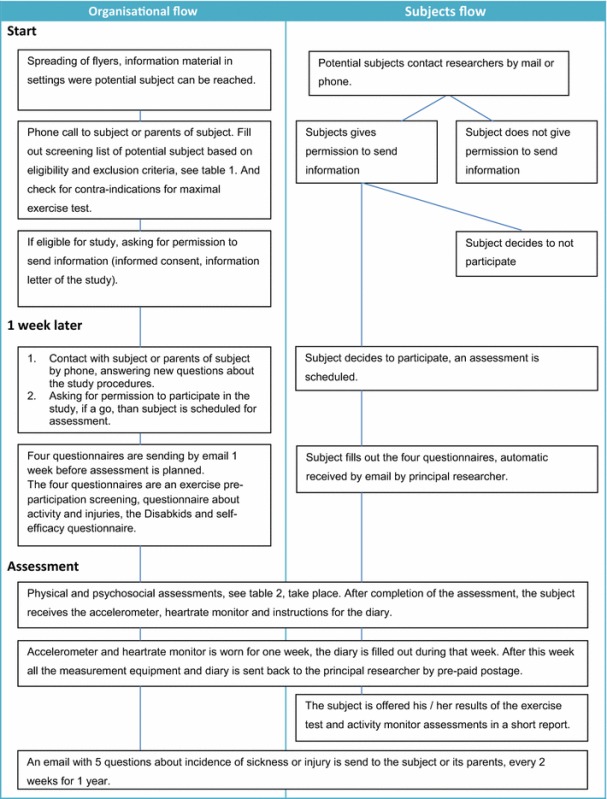
Organisational and subject flow of the HAYS study

### Outcome measures

Table 3 shows an overview of the outcome measures and chosen measurement instruments in this study.

**Table 3:** Overview of assessments used instruments, questionnaires and time schedule

Outcome measure	Parameter	Variable	Measurement	Time at location	Time at home
General	Demographics	DOB, Gender, Diagnosis, FMS score, activity level	General questionnaire		5 min
Cardiovascular health	Metabolic parameters	BMI	Height, Weight	5 min	
		Fat Free Mass	BIA		
		Blood pressure	Sphygmomanometer	10 min	
		Arterial stiffness Pulse wave velocity	Arteriograph		
Physical fitness	Aerobic fitness	VO_2peak_ RER Anaerobic threshold	CPET Shuttle run/ride test Bicycle test	30 min	
	Anaerobic fitness	Peak power Mean power	MPST	5 min	
	Muscle strength	Isometric muscle strength Explosive muscle strength	Grip strength Reverse curl Seated push up Standing broad jump or One stroke push	10 min	
	Flexibility		Modified Apley test Modified Thomas test	5 min	
	Agility		10 × 5 meter sprint	5 min	
Physical activity	Intensity	Heart rate	Actiheart	5 min	7 days
	Modality	Type of activity	Activ8 and Activity diary	5 min	7 days
Cognitive functioning	School performance	Educational level	Type of education Recent CITO-score	0 min	
	Attention	Focused attention Sustained attention Strategy Distractibility	Bourdon-Vos Cancellation task Capture task	15 min 5 min	
Injury and illness	Incidence injury and illness	Retrospective 3 months	Online questionnaire		3 min
		Longitudinal 1 year, every 2 weeks	Online questionnaire		
	Classification of injury or illness (if applicable)		Telephone conversation		5 min
Psychosocial health	Self-perception		Self-perception profile for children (SPPC)	10 min	
	Quality of life		Disabkids		10 min
	Self-efficacy		Exercise self-efficacy Scale		2 min
Total time				150 min	28 min

#### General information

The participants’ characteristics such as date of birth, gender, medical diagnosis, functional mobility scale (FMS) score and type and frequency of sport activity will be identified by a general questionnaire. An exercise pre-participation screening questionnaire will inquire about possible factors influencing the outcomes of the test and the participants’ safety during the tests. These questionnaires will take approximately 5 min to be filled out by the participant and/or parent.

#### Cardiovascular health

Cardiovascular health is divided in several aspects in this study: fat free mass, body mass index, arterial stiffness and blood pressure.

*Fat free mass* will be determined with bioelectrical impedance analysis (BIA), using the Bodystat Quadscan 4000 (EuroMedix, Leuven, Belgium). BIA is a non-invasive easy test to measure lean body mass and fat by comparing conductivity and resistance in the body (Mok et al. 2006). *Body mass index* will be calculated as weight (kg)/height (m)^2^. Weight will be measured using a wheelchair scale in case of subjects who are in the wheelchair group. In other cases a person weighing scale will be used. Height will be measured standing in case of ambulant subjects and supine in case of wheelchair bound persons. In case of spasticity, arm span width will be measured. Arm span length will be measured to the nearest centimetre from middle fingertip to fingertip. Body mass index (BMI) will then be calculated as the body mass divided by the square of arm span length that will be adjusted using arm span ×0.95 for mid‐lumbar lesions and arm span ×0.90 for high lumbar/thoracic lesions in case of central neurological disorders (Dosa et al. 2009).

*Arterial stiffness* has two independent measurement values: augmentation index: (AIX) and pulse wave velocity (PWV). Measurement will take place with the Arteriograph (Arteriograph.nl/LITRA, Amsterdam, the Netherlands). The Arteriograph measures the PWV and the AIX through the brachioradial artery using oscillometric tonometry. Each subject rests supine for at least 10 min before recordings are made. The measurement will take place with an inflatable cuff (similar to blood pressure measurement) at the right upper arm. Instructions to the subject are no food intake 3 h before measurement and no talking during the measurement. *Blood pressure* will also be measured using the Arteriograph within the same measurement.

After the pulse wave velocity measurement, the child is allowed to eat something, before continuing with the rest of the tests.

#### Physical fitness

Physical fitness consists of a combination of aerobic fitness, anaerobic fitness, muscle strength, flexibility and agility. Using the FMS for 500 meters the group of subjects will be divided in two sets depending on their mode of locomotion on this distance: wheelchair users and subjects who are able to walk with or without mobility aids. For these two groups, different testing tools will be used, appropriate for the type of locomotion.

Subsequently, within the group of subjects who are ambulatory different levels of mobility can be identified in order to apply a proper testing protocol, see Table 4.

**Table 4:** Level of mobility for identifying testing protocol

Category	Level of mobility
I	Subjects with a very low level of mobility (able to walk <400 m in 6 min)
II	Subjects with a low level of mobility (able to walk > 400 m in 6 min)
III	Subjects with average level of mobility: walking indoors and outdoors and climbing stairs without limitations and able to perform gross motor skills including running and jumping
IV	Subjects with an excellent level of mobility, who are used to run at a speed of 8 km/h during their practicing their sports or during competition

#### Aerobic fitness

In exercise testing peak, oxygen uptake (VO_2peak_) is considered to be the single best indicator of the cardio-respiratory system, often referred to as aerobic fitness. A cardio-pulmonary exercise testing system, the Cortex Metamax 3X (Samcon bvba, Melle, Belgium), will be used for evaluating the respiratory gasses and VO_2peak_.

Bhambani et al. concluded that maximal exercise testing during the main mode of ambulation elicits the highest oxygen uptake. Therefore the type of sports or daily locomotion determines whether the shuttle run, shuttle ride or a cycling test will be used (Bhambhani et al. 1992).

Children who are able to walk will be tested with an (adapted) shuttle run test (Léger and Lambert 1982; Verschuren et al. 2006). The speed of the shuttle run test will be adjusted based on the results of the muscle power sprint test and the agility test and the level of mobility, see Table 4.

In children with a congenital cardiopulmonary disease a cycling test will be used, because of the monitoring of the heart for safety issues. The cycling test, using the Godfrey protocol (Washington et al. 1994), will also be applied to evaluate the aerobic fitness in children who are active on a bike in sports or daily living. Load depends on height of the child and the expected level of fitness.

A shuttle ride test will be used in children using a wheelchair. Shuttle tests are field tests in which a participant walks or runs between 2 markers. In this case they have to ride a distance of 10 meters in their own wheelchair, if applicable their sports wheelchair, between two cones. The starting speed is 2.0 km/h and the speed is increased with 0.25 km/h every minute. The children have to keep on riding, until they fail to reach the cone two times in a row, despite encouragements. This protocol has been proven valid as a maximal exercise test in youth with CP and spina bifida (Verschuren et al. 2013; Bloemen et al. 2015).

Regardless of the testing modality, the test will start with a resting steady state measurement for 3 min. To reach a total exercise time of at least 6 (children) and 8 (adolescents) minutes the protocol will be adapted based on the expected level of fitness. This might be due to the subjects high competition level for example or extreme athletic physique but also when a very low fitness is expected. In case of the cycling test, an unloaded phase of 1 min will precede the exercise phase. Each test will be until volitional exhaustion. Usual emergency procedures are in place to ensure health and safety in the very unlikely event of an emergency.

#### Anaerobic fitness

The muscle power sprint test is used to measure anaerobic fitness (Verschuren et al. 2007, 2013). Subjects have to complete six 15-m runs at a maximum pace. The MPST is an intermittent sprint test, in which the child stops and starts at standardized intervals. For children who self-propel manual a wheelchair, the MPST has also been proven a reproducible test for measuring anaerobic fitness and agility in children and adolescents with CP and SB (Verschuren et al. 2010, 2013).

Power output will be calculated for each of the six sprints: power = weight × (distance)^2^/(time)^3^. Peak power will be defined as the highest calculated power, while mean power was defined as average power over the six sprints.

#### Strength

To test the strength of the subjects, tests from the Brockport fitness test are chosen (Vanhees et al. 2005).

The handgrip strength will be tested through the use of a hand held hydraulic dynamometer (HHD) as described by Beenakker et al. (2001). The subjects dominant hand will be tested.

To measure the functionality of hand, wrist, and arm the reverse curl is used (Winnick and Short 1999). The participant attempts to pick up a (0.5-kg) dumb-bell with the preferred arm while seated in a chair or wheelchair.

The seated push up test is designed to measure upper-body strength and endurance (Winnick and Short 1999). Participants attempt to perform a seated push-up and hold it for up to 20 s.

The standing Broad jump (only for the ambulatory group) will be used to evaluate the explosive strength of the lower limbs by measuring the distance jumped with two legs together from standing position (Deitz et al. 2007). In wheelchair dependent participants, the one stroke push will evaluate the explosive strength of the upper limbs by measuring the distance one can cover in a wheelchair by one push (Verschuren et al. 2013).

#### Flexibility

To measure upper-body flexibility, the modified Apley test will be applied. The participant attempts to reach back and touch with one hand the superior medial angle of the opposite scapula. The modified Thomas test is designed to assess the length of the participant’s hip flexor muscles (M. Iliopsoas and M. Rectus Femoris) (Winnick and Short 1999).

#### Agility

The 10 × 5 Meter Sprint Test will be used to measure agility (Verschuren et al. 2013). During this test, the child has to sprint (for ambulatory and wheelchair users as well) as fast as possible, 10 times, in between 2 lines that are 5 meter apart. There is no resting period, so the child/adolescent has to turn as fast as possible during this test. Time will be recorded using a stopwatch.

#### Physical activity

Accelerometry in combination with heart rate monitoring will be used to measure the type, duration, frequency and intensity of physical activity in daily life. The Activ8 (2 M Engineering Ltd. Valkenswaard) will measure the modality of physical activity. The Activ8 system is new and has been recently be validated in cooperation with the Erasmus Medial Centre’s rehabilitation department in Rotterdam (Lankhorst K. et al. work in progress). Participants who are able to walk wear one small sensor on the dominant leg. Participants in a wheelchair have to wear 2 small sensors (chest, one wrist). Participants will wear these monitors during the tests and during 7 days in their own environment. Wearing the activity monitor will not alter the activity pattern of the participants; all activities in daily life and in sport can be performed. The Actiheart (Camntech), will be used to detect the intensity of physical activity (Brage et al. 2005). The Actiheart is a small device detecting the heart rate frequency via two electrode-stickers on the chest and has been used previously in children with Spina Bifida (De Groot et al. 2013). During the 1 week of wearing the activity monitor, the participant will also fill out an activity diary in order to interpret missing data of the activity monitor. The completion of the activity diary will only take approximately 5 min per day.

#### Injuries and illness

The incidence and type of injury and illness of the participant at T0 and 3 months in retrospective will be filled out via a digital questionnaire and takes 5 min to complete. The injury density (ID) will be calculated per 1000 h of scheduled physical activity (van Mechelen et al. 1992).

Besides the retrospective registration, the injuries and illnesses will also be monitored longitudinal for 1 year, every 2 weeks. The parent and/or participant receives a short digital questionnaire of 5 simple questions, send by email and takes approximately 1 min to complete. If a question will be answered positively, there will be a telephone conversation with the child, young adult or parent, to detect the cause and severity to register the diagnosis of injury or illness. The researcher will register the injury by using a classification list. Only if the diagnosis remains unclear after the telephone conversation, a physical examination of a sports physician or a physical therapist will take place.

#### School performance

The type of education of the participant will be noted. If available, outcomes of a national educational achievement test (CITO) will be used for quantifying school performance, in both ‘elementary school’ and ‘secondary school’.

#### Cognition

To evaluate cognitive functioning, attention will be measured. To overcome locomotive influences attention tests will be carried out on a tablet (Asus Eee Slate Tablet, with a 12.1 inch display and clock speed of 1.33 GHz).

Three types of attention will be measured: sustained attention, search strategy and distractibility. Sustained attention will be measured using an adapted digitalized version of the Bourdon-Vos task (Vos 1992). This task is a time-limited test. Children had to continue until the whole test was finished, or stop after 10 min. For distractibility an object cancellation task will be used and takes 5 min to complete. The search strategy of the participant during this cancellation test for distractibility is computed afterwards via software of this tablet test. Another test for measuring distractibility is the capture task. This test was adapted from Van Der Stigchel and Nijboer (2010). In each trial the participant is asked to focus on a central fixation cross. When the cross disappears, the target, represented as an apple, appears in one of the corners. In 50 % of the trials, a distractor will appear as well. Reaction time will be measured for both conditions to calculate distractibility.

#### Psychosocial health

To evaluate self-perception in this study, the Dutch translation of the self-perception profile scale (SPPS) for children and for adolescents will be used (Harter S 1985). This questionnaire has been validated to measure self-perception. The questionnaire will take approximately 10 min to complete.

#### Quality of life

To evaluate the quality of life satisfaction, the Dutch version of the Disabkids will be used. This questionnaire measures the quality of life and the independence of children with chronic health conditions. It takes approximately 10 min to fill out.

#### Self-efficacy

To assess self-efficacy specific for exercise and physical activity a Dutch questionnaire is filled out digitally at home by the child and will take approximately 2 min (Nooijen et al. 2013).

## Sample size

The sample size of the HAYS-study is based on a study of Verschuren and Takken (2010). In this study children and adolescents with cerebral palsy had an average peak oxygen uptake of 42 ± 8.2 ml/kg/min. To prove a difference of 10 % between sporting or non-sporting subjects, with an alpha of 0.05 and bèta of 0.20 (power of 0.80) a sample size of 66 subjects per group is required. When taking a failure rate of 10 % into account, 146 subjects should be included in total.

### Statistical analysis

Data will be checked for normal distribution before applying the right type of parametric or non-parametric tests. First, descriptive statistics will be used to describe the two samples. Independent sample T-tests or appropriate non-parametric tests will be used to to determine the differences between the sport and non-sport group.

## Discussion

The current paper describes the rationale, design and methods of a cross-sectional study concerning participation in sports focused on children and adolescents with a physical disability or chronic disease.

To our knowledge this is the first study to evaluate this effect of sport participation on physical, social and mental health within this population. The aim is to determine both the positive and negative effects of sports related to health outcomes in children with disabilities and chronic childhood onset conditions. We hypothesize that the group participating in sports regularly will show higher levels of physical activity associated with better health outcomes, a relative lower incidence density, better cognitive functioning and school performance.

New insights gained from this study could stimulate the improvement of facilities for adapted sports by the government. Also children with a chronic disease or physical disability and their caregivers, sport coaches, physical therapists and other interested parties will be more easily convinced of the advantages of sports participation for this population.

Although we carefully chose our study settings according to its population, we are aware of some limitations of the study.

Objective measurements are chosen where possible. To achieve the best results possible, the best fitting testing modality will be chosen for each participant. The testing modality has to mimic the type of sport participated in. In children who do not participate in sport, the most common form of physical activity in daily life will be chosen as an indicator for the test modality. In general, some test modalities have been proven to elicit a higher VO_2_ peak in the same subject (Bhambhani et al. 1992). The test modality could therefore influence the test outcome. To minimalize this bias we will pursue a comparable number of testing modality types in both groups.

In conclusion, this study will provide insight in the effects of sports participation on the health of children and adolescents with a chronic disease or physical disability.

## Abbreviations

HAYS: Health in Adapted Youth Sports
MPST: muscle power sprint test
VO_2peak_: peak oxygen uptake
BIA: bioelectrical impedance analysis
BMI: body mass index
PWV: pulse wave velocity
AIX: augmentation index
QoL: quality of life
HRQoL: health related quality of life
FMS: functional mobility scale
SPSS: self-perception profile scale
